# Assessing the COVID-19 pandemic’s impact on pediatric baseball and basketball-related craniofacial and neck injuries treated in United States emergency departments, 2003–2022

**DOI:** 10.1371/journal.pone.0302232

**Published:** 2024-04-16

**Authors:** R. Constance Wiener, Eric W. Lundstrom

**Affiliations:** 1 Department of Dental Public Health and Professional Practice, School of Dentistry, West Virginia University, Morgantown, West Virginia, United States of America; 2 Department of Epidemiology and Biostatistics, School of Public Health, West Virginia University, Morgantown, West Virginia, United States of America; Ohio State University, UNITED STATES

## Abstract

**Background:**

A large proportion of United States (U.S.) youth play basketball, baseball, softball, or T-ball. Each of the activities poses a documented risk of craniofacial and neck injuries. However, few studies have assessed the national prevalence of pediatric craniofacial and neck injuries in this population, particularly following the COVID-19 pandemic.

**Methods:**

The National Electronic Injury Surveillance System (NEISS) dataset was used to identify pediatric craniofacial and neck injuries associated with basketball, baseball, softball, or T-ball from 2003–2022 in a cross-sectional study. The annual number of injuries before and after the onset of the COVID-19 pandemic with 95% confidence intervals were calculated. Interrupted time series analysis (ITSA) was used to estimate the pandemic’s impact on the monthly number of injuries incurred nationally.

**Results:**

Both overall and stratified by sport involvement, the annual number and rate of injuries identified in NEISS decreased significantly after the COVID-19 pandemic. ITSA demonstrated that the monthly number of injuries decreased -4094.4 (95% CI = -5100, -3088.7) immediately after the beginning of the pandemic. The number of injuries began increasing towards pre-pandemic levels at a rate of 110.6 (95% CI = 64, 157.2) injuries per month after the initial plunge.

**Conclusion:**

Prior to the-pandemic, there was a steady decline in craniofacial and neck injuries due to basketball, baseball, softball and T-ball among children, aged <18 years. The shutdown during the initial months of the COVID-19 pandemic resulted in a precipitous drop in such injuries. Current rates are approaching pre-pandemic levels and may exceed them. Continued efforts are needed to keep the pre-pandemic progress.

## Introduction

Among U.S. youth, aged 6–12 years, 14.4% played baseball; 14.0% played basketball, and 1.4% played fast-pitch softball in 2019 [1]. Children, less than age 18 years, are vulnerable to injury due to growth and development of cartilage, muscles, and bone, and lack of coordination [2]. Although youth safety in sports has been an important consideration in organized youth sports, it was brought to the forefront by the U.S. surgeon general, in 2000, with strong recommendations for the use of protective gear to prevent sports injuries [3]. Up to that point, one third of all dental injuries were sports related, 41% of baseball injuries involved the craniofacial area [3], and 31% of basketball injuries were orofacial injuries [4]. Following the national call for safety measures, protective equipment was developed and promoted. It has prevented injuries and improved injury outcomes for ball-related injuries [5]. Faceguards were shown to reduce the risk of facial injuries (adjusted odds ratio [aOR]: 0.65) as were safety balls (aOR: 0.77), especially in children aged 7–12 years [5]. These adjustments have reduced acute head and neck injuries as well as the potential long-term or delayed functional, cosmetic, and emotional effects of head and/or neck injuries [6, 7].

There are few current studies providing the prevalence and trends of head and neck baseball-related and basketball-related injuries in youth. In a study using longitudinal Adolescent Brain Cognitive Development 2016–2018 data of children with a baseline age of 9–10 years, 12.2% had lifetime head/neck injuries and participated in non-contact sports as compared with 12.1% of children who had head/neck injuries, but did not participate in non-contact sports (Prevalence rate = 0.966; 95% Confidence Interval, 0.869, 1.07) [8]. In a study of softball injuries for persons aged 7–21 years, the overall national estimate of head/neck injuries was 153,770 (30.2%), which was the most common of the emergency department (ED) softball injuries in this age group from 2010–2019 [9]. Using 2012–2018 National Electronic Injury Surveillance System (NEISS) ED visits for basketball injuries in children aged 7–17 years, researchers found concussion/head injuries occurred in 9.4% of basketball injuries [10]. Most sports injury studies have aggregated overall injuries, or specifically address concussions, a serious condition with increasing surveillance [11]. In a study of sports-related concussions in persons aged 5–21 years, the prevalences were 10.2%, 6.1%, and 3.1% for basketball, softball, and baseball, respectively [11]. In the same study, neck injuries were also reported with prevalences of 3.2%, 3.8%, and 2.1% for basketball, softball, and baseball, respectively [11].

COVID-19, in addition to being highly infectious and resulting in 1.13 million U.S. deaths [12], interrupted economies, schools, workplaces, and organized youth sports in 2020. The disruption in youth sports resulted in parents/guardians not having certain sports available to their child(ren); or considering having their child(ren) quit their sport of interest; or having their child(ren) be involved in a different form of their sport of interest; or in selecting an alternative sport [13]. The post-COVID recovery of organized sports has been innovative and adaptive to meet the changing program preferences, including offering less structure, having programs that are non-league-centered and less rule-bound, and providing drop-in options for participation [13]. With a less competitive emphasis, there may be more children willing to participate for reasons such as spending time with friends and having fun as well as the children whose goals are skills development and exercise [13]. However, parents/guardians in a national study reported having mean scores of 3.76, 3.82, and 3.71 on a scale from 0 (strongly disagree) to 5 (strongly agree) that they were comfortable post-COVID with their child(ren) having pick-up/free play, focused practice/drills, and games/competition, respectively [14]. In a study of ED visits of patients, aged >13 years, a spike in sports-related ED visits post-COVID did not occur despite detrained athletes [15]; however, researchers studying professional athletes did find increased injuries that may have been related to suboptimal sport readiness from home confinement [16]. The post-COVID trends are leading to improvements in sports programs and offerings [13]; however, the impact of the post-COVID period on craniofacial and neck injuries in youth baseball, softball, T-ball, and basketball have not been reported.

The objective of this research is to describe the association of craniofacial and neck injuries requiring ED visits that were associated with youth baseball, softball, T-ball, and basketball activities using NEISS 2003–2023 data in a cross-sectional study design.

## Methods

### Ethical statement

This research was acknowledged as non-human subject research by the West Virginia University Institutional Review Board (protocol 2103254382) and conforms to the Declaration of Helsinki.

### Data source

The data for this research were from queries of the NEISS query builder for the years 2003–2022 among children aged <18 years [17]. The data inclusion criteria for this study were reports of children, aged <18 years, who had an ED visit with craniofacial or neck injuries associated with a basketball or baseball from 2003 to 2022 using the NEISS data. The NEISS database is a publicly available data source available at the NEISS website, https://www.cpsc.gov/cgibin/NEISSWuery/home.aspx. It has a representative sample of 100 ED to create a nationally representative estimate of product-related injuries in the U.S. For this study, baseball, softball, and T-ball activity, apparel, or equipment (code 5041, referred to as “baseball” throughout this article) and basketball activity and related equipment (code 1205) related injuries to the head or neck were extracted. Selected NEISS codes for the head and neck included head code, 75; face code, 76; eye code, 77; mouth code, 88; ear code, 94; and, neck code, 89. Injury types included were: avulsion code, 72; concussion code, 52; contusion or abrasion code, 53; crushing code, 54; dental injury code, 60; fracture code, 57; hematoma code, 58; hemorrhage code, 66; inter-organ injury code, 62; laceration code, 59; nerve damage code, 61, puncture code, 63; strain/sprain code, 64; other code, 71; and not stated or unknown code, 70. For NEISS data to be considered reliable, the coefficient of variation should be <33%, the population estimate should be >1,200, and the number of records should be > 20 [17]. The extracted injuries for 2003–2022 met these criteria. Data for population estimates for children aged <18 years were from the U.S. Census Bureau, provided by the Federal Interagency Forum on Child and Family Statistics [18].

### Statistical analysis

All analyses were performed in RStudio version 4.2.2. The annual number and rate at which injuries were incurred for the years before (2003–2019) and after (2020–2022) onset of the COVID-19 pandemic was estimated using the R packaged ‘survey’ and ‘srvyr’ with product codes for both basketball-related injuries and baseball-related injuries and stratified by basketball-related injuries and baseball-related injuries.

Interrupted time series analysis (ITSA) was used to quantify the impact of the COVID-19 pandemic on monthly craniofacial and neck injuries captured in NEISS. ITSA measures the impact of natural events by segmenting time series data into pre- and post-intervention periods and is therefore useful in situations in which randomized controlled trials are not feasible. As the intervention assessed was the COVID-19 pandemic in the US, the pre-intervention period for this study was defined as January 2002-March 2020 and the post-intervention period was defined as April 2020-December 2022. The pre- and post-intervention periods were assessed using the model:

$$y_{t}=\beta_{0}+\beta_{1}t+\beta_{2}P+\beta_{3}D+\epsilon$$

where *y*_*t*_ is an outcome of interest, *β*_0_ is the model intercept, *t* is time, *P* is a variable representing time since the intervention (zero before the intervention, slope of one afterwards), and *D* is a dummy variable representing the immediate effect of the intervention [19, 20]. *β*_1_, *β*_2_, and *β*_3_ represent the pre-intervention slope, the sustained post-intervention effect (i.e., a slope change impact known as a “ramp” variable), and the immediate post-intervention effect of an intervention (i.e., a “step-change” impact), respectively. Model error is denoted by *ϵ* and, in the case that serial data correlation is present (i.e., annual seasonality) and the linear regression assumption of data independence is therefore violated, includes autoregressive integrated moving average (ARIMA) terms. The need for ARIMA errors terms was assessed via visual inspection of model residuals via autocorrelation plots and non-significance of the model’s Ljung-Box test.

## Results and discussion

The total number of basketball and baseball-related pediatric craniofacial and neck injuries for 2003–2019 was 1,408,003. The mean annual basketball and baseball-related pediatric craniofacial and neck injuries for those 16 years was 88,000.1875 (95% CI = 75,487; 100,514) and represented 112.4 (95% CI = 96.4; 128.4) such injuries per 100,000 U.S. population aged <18 years (Tables 1 and 2).

**Table 1:** Number of craniofacial and neck injuries (95% confidence interval) associated with basketballs and baseballs in the U.S. population, aged <18 years.

Category	2003–2019	2020	2021
	Number of injuries	Number of injuries	Number of injuries
	(95% CI)	(95% CI)	(95% CI)
**Total basketball (1205) and baseball (5041) craniofacial and neck injuries**	1,408,003	28,201	36,612
	(1,207,788; 1,608,218)	(21,732; 34,671)	(25,449; 47,775)
**Basketball (1205) craniofacial and neck injuries**	791,243	18,726	19,391
	(677,863; 904,624)	(14,439; 23,012)	(14,080, 24702)
**Baseball (5041) craniofacial and neck injuries**	616,760	9,476	17,221
	(520,221; 713,299)	(6,626; 12,316)	(11,053; 23,389)

Basketball craniofacial and neck injuries were identified with NEISS code 1205. Baseball craniofacial injuries were identified with NEISS code 5041. Columns indicate the number of such injuries and the 95% confidence interval.

**Table 2:** Craniofacial and injuries (95% confidence interval) per 100,000 U.S. population, aged <18 years, associated with basketballs and baseballs.

Category	2003–2019	2020	2021
	Injuries per 100,000	Injuries per 100,000	Injuries per 100,000
	(95% CI)	(95% CI)	(95% CI)
**Total basketball (1205) and baseball (5041) craniofacial and neck injuries**	112.4	38.0	49.8
	(96.4; 128.4)	(29.3; 46.7)	(34.6, 64.9)
**Basketball (1205) craniofacial and neck injuries**	63.2	25.2	26.4
	(54.1; 72.2)	(19.5; 31.0)	(19.1; 33.6)
**Baseball (5041) craniofacial and neck injuries**	49.3	12.8	23.4
	(41.5; 57.0)	(8.9; 16.6)	(15.0; 31.8)

Basketball craniofacial and neck injuries were identified with NEISS code 1205. Baseball craniofacial injuries were identified with NEISS code 5041. Columns indicate the number of such injuries per 100,000 U.S. population, aged <18 years and the 95% confidence interval.

Pre-COVID estimated monthly basketball and baseball-related pediatric head and neck injuries from January 2023 to March 2020 (before the sentinel COVID-19 event) decreased at a rate (slope) of -11.4 (95% CI = -18.6; -4.1) per month (Table 3).

**Table 3:** Rate of monthly craniofacial and neck injuries in children, aged <18 years, associated with basketballs and baseballs.

Category	Value	95% Confidence Interval
**Total basketball (1205) and baseball (5041) craniofacial and neck injuries**		
**Pre-COVID slope (2003–2019)**	-11.4	-18.6; -4.1
**Immediate impact of COVID-19(2020)**	-4,094.4	-5,100.0; -3,088.7
**Post-COVID slope (2021–2022)**	110.6	64.0; 157.2
**Basketball (1205) craniofacial and neck injuries**		
**Pre-COVID slope (2003–2019)**	-2.0	-6.2; 2.3
**Immediate impact of COVID-19 (2020)**	-2,616.0	-3,290.9; -1941.2
**Post-COVID slope (2021–2022)**	48.0	16.4; 79.6
**Baseball (5041) craniofacial and neck injuries**		
**Pre-COVID slope (2003–2019)**	-10.1	-16.9; -3.3
**Immediate impact of COVID-19 (2020)**	-1,289.4	-1,822.6; -756.1
**Post-COVID slope (2021–2022)**	61.4	34.7; 88.0

Basketball craniofacial and neck injuries were identified with NEISS code 1205. Baseball craniofacial injuries were identified with NEISS code 5041. The results indicate the time trajectory rate (slope) before the sentinel event (COVID-19) which were negative, indicating the number of injuries were decreasing, the impact of COVID-19, which was a rapid decrease, and the trajectory rate (slope) was an increase toward the pre-pandemic level after the sentinel event.

**Fig 1:**
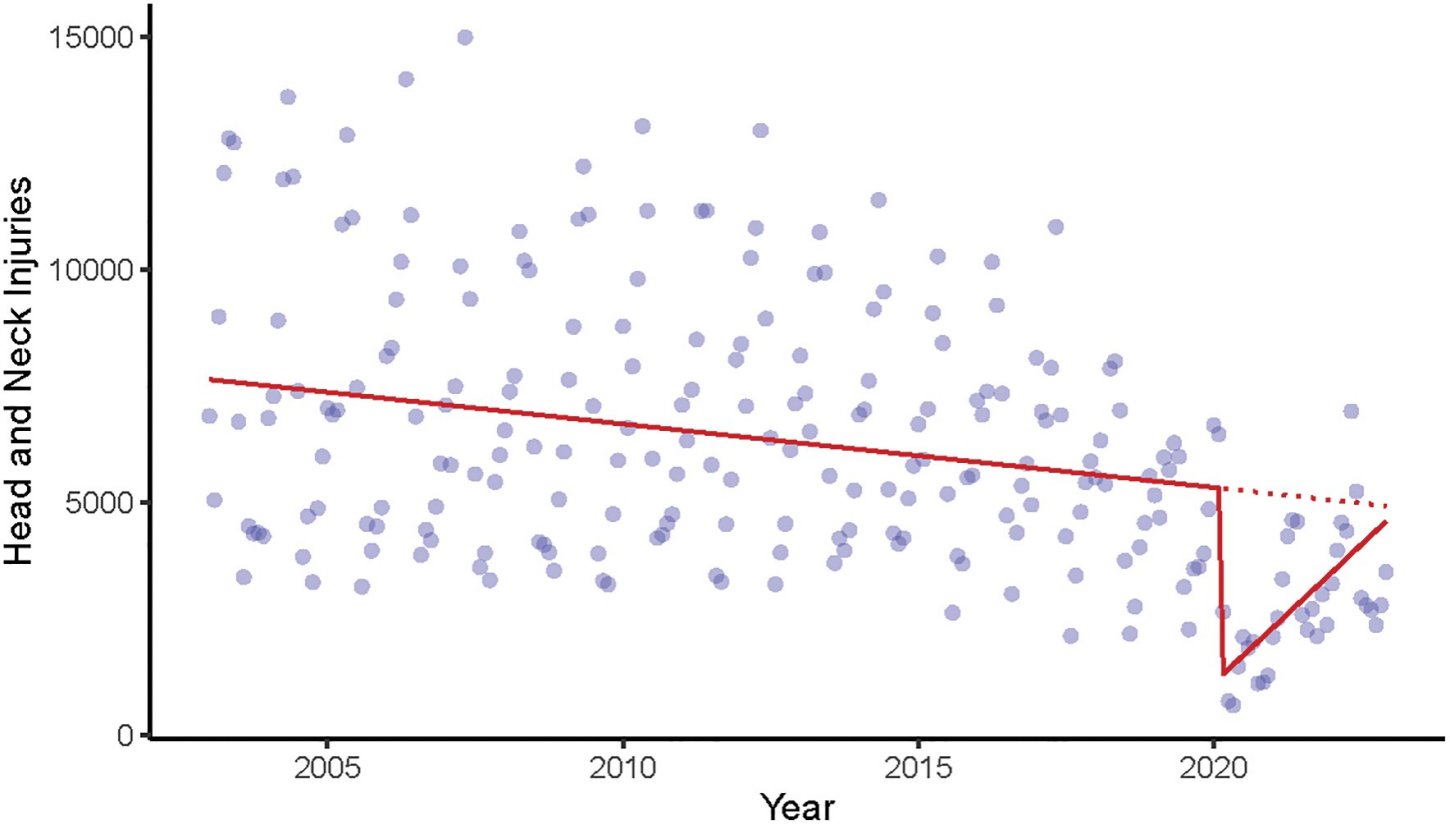
Number of craniofacial and neck injuries (95% confidence interval) associated with basketballs and baseballs in children, aged <18 years. Plot of craniofacial and neck injuries associated with basketballs and baseballs from 2003–2022 in children, aged <18 years. The red line indicates the time trajectory (slope) associated with the injuries. The dotted red line indicates the expectation of the trajectory. The plunge below the dotted red line indicates the impact of the sentinel event, COVID-19.

ITSA of total monthly basketball and baseball-related pediatric head and neck injuries are depicted visually in Fig 1.

Immediately following the beginning of the COVID-19 pandemic, the mean number of total monthly injuries incurred dropped drastically to -4094.4 (95% CI = -5100, -3088.7) lower than pre-pandemic. An equally rapid increase towards pre-pandemic levels occurred at a rate of 110.6 (95% CI = 64.0, 157.2) injuries per month after the initial plunge in 2020; as shown in Fig 1. Monthly injuries had not reached pre-pandemic levels (red dotted line on Fig 1) by the end of 2022. ITSA stratified by product code showed similar patterns: decreasing monthly injuries pre-pandemic, an immediate drop following COVID-19 onset, and a slow return towards pre-pandemic levels. However, the rate at which basketball-related injuries decreased pre-pandemic was not significant (-2.0 per month; 95% CI = -6.2, 2.3).

The number of overall craniofacial and neck injuries in youths participating in baseball, softball, T-ball, and basketball decreased from 2003 to 2019. A significant, precipitous drop in injuries was detected both via annual and monthly analysis, corresponding with COVID-19 lockdown and restrictions. In the months that followed (April 2020-December 2022), there was a slow rise towards pre-pandemic levels. However, injuries had not yet reach pre-pandemic levels by the end of 2022. There was no significant difference in the number or rate of injuries incurred among product codes (i.e., basketball vs baseball-related). In 2020, basketball-related injuries were incurred at a rate significantly greater than baseball-related injuries; however, no significant difference by product code was detected in 2021 or 2022.

There were many factors that could have affected the number of craniofacial and neck injuries in children due to baseball, softball, T-ball, and basketball activities during the pandemic. Fewer injuries would have resulted from fewer sport-related occasions. From 2019 to 2020, the number of occasions basketball was played by children, aged 6–12 years, decreased 3.6% (479 billion times to 433 billion times); baseball decreased 9.5% (479 billion times to 433 billion times); and softball decreased 5.2% (120 billion times to 114 billion times) [1]. In addition to the number of occasions that decreased, the number of children, aged 6–12 years, playing baseball dropped 15.2% to 3,403,000 in 2020 (Project Play, 2020). The number of children, aged 6–12 years, playing softball dropped 16.6% to 331,000; however, the number of children, aged 6–12 years, playing basketball increased 5.5% to 4,114,000 [1]. Additionally, fewer such injuries may have occurred during the early phases of the pandemic to children in families that had the financial ability and access to pay for private coaching of their child/children, or who had coaches that provided online workout routines that kept the youth active and in fit physical conditions [21].

On the other hand, there were also increased risks during the return-to-play. During the early phases of the COVID pandemic, the shutdown of schools resulted in youth losing opportunities to participate in sports, increasing risks in return-to-play [22]. There were increased bone, ligament, and cartilage risks identified in youth due to pandemic-related deconditioning, obesity, lack of variety of physical activities with growth centers of bones being at increased risk in deconditioned athletes with abrupt return-to-play [22].

Another factor for the number of craniofacial and neck injuries in children due to baseball, softball, T-ball, and basketball was family finances. COVID changed many family perspectives about their child’s or children’s sports. Before the pandemic, many families centered their time, resources, and family activities around their child’s or children’s sports. Children’s sports activities influenced family meals, family travel, and family vacations [21]. Many families aspired for their children to receive athletic college scholarships [21]. COVID impacted many families’ discretionary income over the long-term. Their ability to purchase the needed equipment (including protective equipment), travel, league memberships, etc. affected return-to-play [21] with many children not returning to their sport. Thus, financial hardship decreased the number of sport occasions [23], and therefore decreased the (counterfactual) number of injuries.

An often-overlooked sports injury consideration is illegal/foul play activity. Little research is available about the potential impact of COVID-19 on illegal/foul play activity. In Pre-COVID high school play (2005–2007), researchers reported 6.4% (98,066) high-school sports injuries in football, basketball, wrestling, baseball, softball, girls’ soccer and volleyball (0.24 injuries per 1000 competition-exposures) were due to illegal/foul play and 32.3% of these were to the head/face.^24^ The authors recommended enhanced enforcement of existing rules as well as player, coach, referee/official education about the consequences of illegal/foul play [24]. In a Pre-COVID versus COVID-19 comparison study of basketball performance, personal fouls, though not the focus of the study, were included in the study. Pre-COVID matches (with spectators) had a significant difference in fouls between those made by away teams and home teams with away teams using fouls to disrupt game pace and to be more aggressive; however, during COVID-19 when matches did not have spectators, there was no significant difference in fouls made between home and away teams [25]. Mention was not made in the research as to the relationship of the fouls to injuries. In a combined Pre-COVID/COVID era qualitative study of middle school athletes’ parents, it was noted that pressure to win may lead to [home] officials to miss illegal/foul play, and children with injuries may feel pressure to continue with play and not report injuries [26]. Sports culture has a role in pediatric sports injuries.

### Strengths and limitations

This study includes ten years of national data in describing the trend of craniofacial and neck injuries in children in the U.S. The data are current and explain an important issue affecting many children in the U.S. and national injury estimates are possible. The data are the result of reports from emergency departments in which the reporters have described injuries to the head and neck from baseball, softball, T-ball, and basketball activities. As the data are not collected specifically for research, there is the potential for underreporting bias. Also, injuries that did not require emergency department care, or were treated in an urgent care facility or dental office, are not reflected in the study.

### Policy considerations

Injury prevention and risk reduction are important in pediatric sports. There is a need for protective equipment such as faceguards and safety balls in pediatric sports [27]. Current baseball rules for youth require batters to wear a batter’s helmet with a double earflap while batting, on deck, and running the bases. Catchers must wear a catcher’s helmet and facemask as well as a throat guard. Optionally, elbow protection is permitted and defensive players may wear non-glare face/head protection in the field [28]. The ADA Council on Scientific Affairs and the ADA Council on Scientific Access, Prevention and Interprofessional Relationships recommends the use of mouthguards in contact and collision sports [29]. Its website provides a list of 12 contact/collision sports, including basketball, and 17 limited contact sports, including softball and baseball at its website for mouthguard use recommendations [29]. The dental profession has a role in directing the course of the sports culture in accepting and using properly fitted mouthguards to limit concussions and dental injuries.

## Conclusion

Many factors were involved in the steady decline in basketball, baseball, softball and T-ball craniofacial injuries in children <18 years since 2003. The shutdown of the initial months of the COVID-19 pandemic resulted in an associated precipitous drop in such injuries. Since the initial drop, current rates are approaching pre-pandemic levels and may exceed them. There is a need for stakeholders to continue the progress toward decreasing the number of injuries associated in youth basketball, baseball, softball, and T-ball activities.

## Supporting information

S1 File Basketball and baseball injury data. (XLSX)
